# Occupational and personal factors contributing to participation in occupational health promotion programs for employees in nursing home facilities: a secondary analysis of the PROCARE study

**DOI:** 10.1186/s12912-026-04973-6

**Published:** 2026-07-09

**Authors:** Tanja I. Janssen-Masmeier, Oliver Vogel, Ann-Kathrin Otto, Thomas Klotzbier, Heide Korbus, Luca Antonia Grühn, Claudia Hildebrand, Thomas Jöllenbeck, Daniel Schoene, Nadja Schott, Lutz Vogt, Matthias Weigelt, Claudia Voelcker-Rehage, Bettina Wollesen

**Affiliations:** 1 Department of Neuromotor Behavior and Exercise, Institute of Sport and Exercise Sciences, University of Münster, Wilhelm-Schickard-Straße 8, 48149 Münster, Germany; 2 Department of Human Movement Science, University of Hamburg, Hamburg, Germany; 3 Department of Sport and Exercise Science, University of Stuttgart, Stuttgart, Germany; 4 Karlsruhe Institute of Technology, Karlsruhe, Germany; 5 Department of Sport and Health, University of Paderborn, Paderborn, Germany; 6 Friedrich-Alexander-University of Erlangen-Nürnberg, Erlangen, Germany; 7 Department of Sport Medicine, Goethe University Frankfurt, Frankfurt am Main, Germany; 8 Chemnitz University of Technology, Institute of Human Movement Science and Health, Chemnitz, Germany; 9 JICE, Joint Institute for Individualisation in a Changing Environment, University of Münster and Bielefeld, Münster and Bielefeld, Germany; 10 Institute of Movement Therapy and Movement-Oriented Prevention and Rehabilitation, German Sport University Cologne, Cologne, Germany

**Keywords:** Occupational health promotion, Participation, Nursing staff, Long-term care, Workplace, Health intervention, Coping behavior, Occupational stress, Health management

## Abstract

**Background:**

Nursing staff are exposed to demanding occupational conditions, including physical load, physiological stress and negative institutional factors that are associated with job turnover and unhealthy behavior, health complaints (such as back pain), and other issues. Occupational health promotion programs, such as ergonomics and exercise, are offered to counteract these unfavorable trends, but participation is often low. However, knowledge about factors favoring or reducing the participation of nursing staff in occupational health promotion programs in nursing home settings remains incomplete. Given this limited and inconclusive evidence, this study followed an exploratory approach for the participation analysis.

**Methods:**

This cross-sectional secondary analysis explored potential predictors that may be associated with participation in tailored occupational health promotion programs in nursing home facilities. We analyzed multicenter data of nurses (*n* = 310) from 47 nursing homes invited to participate in tailored occupational health promotion programs (PROCARE study). We focused on reasons for (non-)participation, including occupational and individual factors and conducted a logistic regression analysis to examine the potential predictors associated with nurses’ participation in occupational health promotion programs.

**Results:**

The findings suggested that a better relationship with colleagues (workplace environment) (OR = 0.569; CI [0.360 0.899]) and a higher physical activity level (individual health behavior) (OR = 0.525; CI [0.328 0.842]) were associated with a higher likelihood of participation in occupational health promotion programs.

**Conclusions:**

We identified possible predictors associated with participation, such as the work environment and the individual health behavior, that could be considered during the planning and implementation of occupational health promotion programs. Future programs might consider a linkage to individuals’ health behavior change strategies and address the relationship to colleagues. This tailoring could be particularly relevant to support employees’ participation in occupational health promotion programs in the implementation phase.

**Trial registration:**

The PROCARE study was registered with the German Register of Clinical Trials (DRKS.de; DRKS00015241).

**Supplementary information:**

The online version contains supplementary material available at 10.1186/s12912-026-04973-6.

## Background

In line with the aging population, the number of people depending on long-term care is rising [1]. For example, the number of Germans requiring nursing care is estimated to increase to 7,6 million in 2050 [2]. In December 2024, about 800,000 nursing home staff were available [3]. Along with a growing number of residents, the shortage of personnel and high turnover rates lead to an imbalance between staff and residents, which underscores the importance of promoting employee health. Moreover, the occupational conditions in the nursing sector are characterized by high physical loads [4] and psychological stress [5] as well as unfavorable institutional factors and often inappropriate personal health behaviors or selfcare [6, 7].

### Occupational health promotions programs for nursing staff

To improve the health of nursing staff, occupational health promotion programs often focus on ergonomics, strength training [8], or other forms of exercise [9]. Other address psychological demands, stress and coping strategies and individual health behavior like nutrition [10]. A systematic review revealed nutrition interventions in the occupational setting to be most effective, followed by body composition, physical activity, and stress interventions, and interventions mainly focusing on health outcomes (e.g. well-being) being less effective. Moreover, addressing the behavioral change processes rather than education appears to be more favorable to improve participation [11]. In addition, employee involvement and engagement in the implementation process (integration) seem to be required to support participation. This also enables tailoring of the intervention to the specific needs of the nursing staff [12].

A theoretical framework for the design and implementation of occupational health programs that considers all these aspects, including behavioral change and employee involvement, is **BASE** [13]. The BASE Model is a theoretical framework combining physical and psychological health dimensions and is theoretically based on the Health Action Process Approach (HAPA) model [14] and the PRECEDE-PROCEED model [15]. The four components of BASE are i) needs assessment, (ii) workplace organization and participatory decision on interventions, iii) coaching preventive behavior at work, and iv) empowerment and evaluation [13]. The “needs assessment” typically relies on standardized questionnaires collecting physical and psychological load and demands, work environment and health behavior, reflecting the requirements, wishes and strains of the employees in the occupational field. Based on the results of the needs assessment, tailored programs are derived (ii) and the implementation process for tailored interventions is discussed with the organization using a participatory approach (iii). The final step (iv) of BASE is the evaluation of the developed and implemented program with respect to participation, satisfaction and health effects.

### Participation and their factors in occupational health promotion programs

Participation can be regarded as an important indicator of implementation success [13, 14, 16]. ‘Participation’ refers to attendance in a course, training or program. Participation rate reflects the intensity and/or extent of participation. The assessment of participation in occupational health promotion programs is relevant for determining their cost-effectiveness and health-effectiveness, as the health-related effectiveness of a program is influenced by participant attendance [17, 18].

Interestingly, many intervention studies do not report participation or report rather low participation rates. For example, participation rates in movement-related programs range from 18% to 36%, depending on program type and cohort [19, 20], in stress reduction programs, they are up to 80% [21]. Moreover, women are generally more likely to participate in health promotion programs than men [19]. In Germany, about 50% of the companies offer occupational health promotion programs and report participation rates of about 15 to 25% [18, 22]. Also, high drop-out and/or low compliance for occupational health promotion programs, especially in small companies, were noticed [23, 24].

Participation is determined by different factors as detailed below. Occupational and individual factors, such as physical workload, psychological demands, stress and coping strategies and individual health behaviors may influence participation as well as motivation, perceived additional value, social support, and time resources [25, 26]. Other factors that might facilitate participation are a good marketing and information flow, a program that is close to the workplace and considers the needs of the employees, an implemented infrastructure and a realistic schedule [27]. However, evidence on the impact of these factors on participation in tailored occupational health promotion programs in nursing homes is limited [28].

#### Participation and physical workload in nursing staff

The physical demands of nursing staff include lifting and repositioning patients [29] as well as standing for long periods [30]. The resulting physical load has been identified as a risk factor for musculoskeletal disorders [31]. Common musculoskeletal disorders include neck and back pain and often lead to sick days [31–33]. However, the role of these factors for participation in occupational health promotion programs in nursing home settings remains unclear [34].

#### Participation and psychological demands, stress, and coping strategies in nursing staff

Nursing staff also experience high levels of psychological stress [35, 36]. Time pressure, resulting from staff shortages [37], is a key stressor and negatively affects care quality [38]. Additional stressors include responsibility for individuals in need of care, high-quality expectations, low decision-making authority, high workload, role conflicts, and exposure to aggression [34, 39]. Elevated psychological stress is associated with psychological impairments such as fatigue [40] and depression [41, 42]. Identified occupational coping strategies, including overexertion and resignation, may influence stress perception, sickness absence [43], and overall health [44]. Furthermore, low perceived occupational appreciation and unfavorable coping strategies may increase job turnover [45]. Occupational psychological demands and stressors may also be associated with participation in occupational health promotion programs [46].

#### Participation and work environment in nursing staff

In addition, aspects of the work environment, including leadership and communication style, could burden nursing staff and their health status [47], and probably influence participation. Leadership style and employee relationships are associated with work-related well-being and job turnover rates [6, 48]. The same applies for low levels of job control and co-determination [49] as well as high job demands [50]. Conversely, good communication and work climate are associated with healthy, satisfied nursing staff [51], and team cohesion [52]. Accordingly, these factors may also affect participation, although this relationship has not been analysed for occupational health promotion programs in nursing homes yet.

#### Participation and individual health behavior in nursing staff

Also, the individual health behavior such as physical activity or nutrition behaviors may extend to the occupational setting [53] and may influence participation. In this vein, it has been shown that the participation in occupational health promotion programs for hospital nursing staff seems to be positively associated with practicing positive healthy behaviors [54]. However, nurses often show negative health behaviors [55]. These include improper lifting and carrying [56, 57], smoking [58, 59], as well as poor lifestyle behaviors, such as low physical activity during leisure-time [60] and inadequate nutrition habits [59, 61]. Whether general health lifestyle or one specific health behavior (only one behavior) in nursing staff affects participation remains unclear.

### Present study

The present study aimed to identify factors that influence participation in a tailored occupational health promotion program following the BASE framework among nursing staff in nursing home facilities. Therefore, we analysed the interplay between occupational and individual factors. Occupational factors comprised physical, psychological, and environmental factors, whereas individual health behaviors (i.e., physical activity behavior, nutrition) were considered as individual factors. Based on the current state of knowledge, we expected that participation in occupational health promotion programs is associated with 1) the physical load, 2) the perceived psychological demands, stress and coping strategies, 3) the work environment, and 4) the individual health behavior.

## Method

### Study design

This secondary analysis was based on data from the project ‘PROCARE – Prevention and occupational health in long-term care’ [62]. The PROCARE study is a randomized controlled trial (RCT), that was conducted in 47 nursing home facilities in Germany between April 2017 and March 2020. This primary study was approved by the local ethics committee of the University of Hamburg (AZ:2018_168) and is registered with the German Register of Clinical Trials (DRKS.de; DRKS00015241). In this study, a cross-sectional analysis was conducted on the RCT-PROCARE data set. More information on recruitment and enrolment can be obtained from the study protocol [62].

### Study population and sample size

**Fig. 1:**
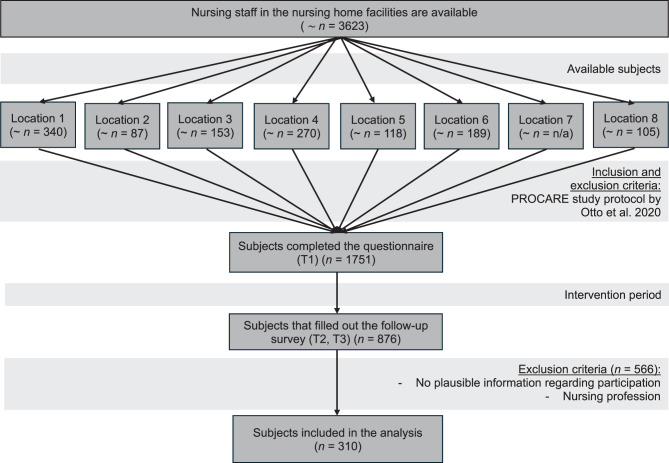
Flow chart of the data extraction

All employed nurses *(N* = 3623) in the participating nursing homes were offered to the opportunity to attend comprehensive information events on occupational health promotion programs. One thousand seven hundred and fifty-one participants agreed to participate and signed a written informed consent aligned with the principles of the Declaration of Helsinki (2013) and filled out the first questionnaire. Eight hundred forty-six subjects filled out questionnaire 2 resp. 3. Participant selection and eligibility criteria for the current analysis of cross-sectional data were based on the primary outcome participation in the PROCARE study [62]. Due to missing information about their participation, 536 participants were excluded from the final analysis. In the group comparison and logistic regression, we integrated *n* = 310 participants (mean age = 42,3, standard deviation = 11,7; female = 239). The extraction process for the final dataset is shown in the flowchart in Fig. 1. The following variables were selected and defined based on the theoretical premise of the BASE framework.

### Participation (Dependent variable) (BASE - Step 4)

The primary outcome of this study was the rate of participation in the occupational health promotion programs (ergonomics and back fitness). Participation was assessed in a follow-up survey by using a single self-report question (“Did you participate in the occupational health promotion program?”). Based on the question, the participants were divided into two groups: participation (*yes, coded as 2*) and non-participation (*no, coded as 1*)(revised-coding). As participation in the occupational health promotion program was only assessed in the follow-up survey, participants who did not complete this survey were considered non-participants. The following components are sub-elements of the results that were developed and collected as a result of the structure of the PROCARE study [62].

### Possible predictors and measurements (BASE - Step 1)

The possible predictors were derived from the BASE framework step 1 need assessment and were organised into conceptual domains for analytical clarity, including physical load and psychological demands, stress level, coping strategies, health status, work environment and individual health behavior. This organisation reflects the conceptual circular approach, not a hierarchical order of predictors, as within the BASE framework the components have no hierarchical order.

#### Physical load and psychological demands

The *Questionnaire for Subjective Assessment of Workplace Exposure* (modified FEBA Questionnaire) evaluates work-related demands (physical, psychological, and environmental factors) and the respondent’s perceived exposure to them [63]. The questionnaire is a validated and reliable tool [64]. Participants rate the frequency of demanding factors (frequently, seldom/never) and the amount of physically or mentally perceived exposure (yes, no) dichotomously [64]. After factor analysis, we identified two factors: physical demands with 6 components (holding, carrying, lifting, pulling/pushing, heavy loads, heavy physical work) and psychological demands with 3 components (pressure to perform, deadline pressure, time pressure) [44]. Two sum scores were formed based on the occurrences of burden in these components. The physical demands score ranged from 0 to 6, and the psychological demands score from 0 to 3. All items were equally weighted in their score. Higher scores indicate higher burden in physical demands or psychological demands. Higher values in both scores represent higher physical and physiological demands. Scores were computed only when no missing values were present for a subject, because of a simple additive scoring system (complete cases calculation). This standardized scoring approach and the reduction of the items was developed in the PROCARE study and has been applied in previous research [44, 64, 65].

#### Stress level

Overall stress level was assessed using the German version of the 12-item screening subscale (SSCS) of the *Trier Inventory for Chronic Stress* (TICS [66, 67]). The questionnaire measures stress, chronic worrying, lack of social respect, job-related and social overload, and excessive demands. It describes 12 items that should be rated on a five-point Likert scale (0 = never, 1 = rarely, 2 = sometimes, 3 = often, 4 = very often). The SSCS score ranges from 0 to 48 points and higher values represent higher stress perception [66, 67]. The total SSCS sum score was calculated only if no more than three items were missing according to the scoring recommendation [68] and was used for the present dataset and represents the perceived stress. The internal consistency (Cronbach alpha) is good, with a range of 0.84 to 0.91 [66].

#### Coping strategies

The *Questionnaire on Work-Related Behavior and Experience Patterns* (AVEM) is a multidimensional personality diagnostic tool comprising 44 items [69]. It assesses self-reported health status, resources, and job demands among nursing staff. The AVEM measures 11 dimensions that can be grouped into three major content areas: professional commitment, resistance towards stress, and emotional well-being, all within the work context. Based on the respondents’ ratings of the 44 items, their work-related behavioral pattern is categorized as one of four coping types: Pattern G [**G**esundheit], “health”, Pattern S [**S**chonung] “unambitious”, Risk Pattern A [**A**nstrengung], “Overexertion”, and Risk Pattern B [**B**urnout], “Burnout”. Using the computer software provided by the AVEM authors, a cluster analysis was conducted for every participant to rate the fitting in the predefined pattern types. A clear sorting into one pattern type was possible if the fit exceeded 50% and no other pattern expression was above 30%. For each pattern, a percentage score was computed indicating the degree to which a participant matches the pattern. Higher percentiles represent a higher match with the respective coping pattern. This score was used as a potential predictor in the regression analysis. Cronbach alpha ranged between 0.75 and 0.84 [69]. In consultation with the developer of the questionnaire, the approach to handling missing data was evaluated as part of the primary study. When one missing value in one of the four scales occurs, the missing item was replaced by the other three items of the scale [44]. Imputation was based on the other three scale items to enable the coping type pattern assignment. If more than one value in the four scales was missing, no coping pattern assignments were determined for that respondent [44].

#### Health status

The 12-item *Health Survey* (SF-12) was used to measure health-related quality of life in physical and psychological health dimensions [70]. Within this study, the SF-12 is used as an overarching indicator of subjective physical and psychological health components. Physical and psychological health subscales were measured, each based on four items. Physical health summarizes answers related to physical functioning, role limitations due to physical health problems, pain, and general health. Psychological health summarizes answers related to vitality (energy/fatigue), social functioning, role limitations due to emotional problems, and psychological health (psychological distress and psychological well-being) [70]. For this questionnaire, Cronbach alpha ranged between 0.57 and 0.94 [71]. The two scales of the SF-12 (physical and psychological health), which range from 0 to 100 points, were utilized in our analysis. Higher values represent better health status in the subdimensions. If any values were missing, the SF-12 was calculated using the EM-estimator method based on the existing values as recommended in Wirtz et al. [72].

#### Work environment

The *Questionnaire on Work Environment* was developed by the PROCARE team in cooperation with the nursing home facilities^1^. Participants indicate their responses on a six-point-Likert-scale, from true to not true, to statements in three categories: seven statements relating to colleagues, for example, “If someone has difficulties at work, he/she will certainly be helped by colleagues.”; nine statements relating to supervisors such as “Supervisors respond to our concerns and complaints.”; and six statements in the areas of information and communication exchange, such as “We are sufficiently informed about important things and processes in our company.” The mean value for the three categories was calculated and used as a score if less than 10% were present. A higher score ranging from 1 to 6 represents a better work environment in the respective category. Out of this questionnaire, we used the categories colleagues, supervisors, and information structure and co-determination as parameters representing the work environment. The questionnaire has been reviewed by an expert group for occupational health in nursing home settings. Other validation or reliability testing was not performed on the questionnaire.

#### Individual health behaviour

The *Questionnaire on Resources* (also developed by the PROCARE team according to WHO recommendations [73]) assesses respondents’ health behavior based on the type and duration of weekly and daily activities (physical activity), their nutrition, and stress-balancing activities (stress)^2^. A mean score was calculated based on the following questions: “How many hours per week do you spend exercising?” and “How many minutes per day do you walk or ride a bike?” for the personal health behavior physical activity; “How would you rate your eating habits (healthy-unhealthy)?”, “How many days per week do you have breakfast?” and “How many days per week do you eat fruit and vegetables?” for the health behavior nutrition; “How many times have you been calm and serene in the past four weeks?”, “How many times have you been full of energy in the past weeks?” and “How high would you rate your personal stress level?” for the health behavior stress. The questions are derived from validated and reliable questionnaires, including the Health Survey (SF-12) [71], the Bone-Specific Physical Activity Assessment Instrument [74], and the WHO guideline ‘Sugars intake for adults and children’ [75]. Each scale ranges from 1 to 5, with higher values representing better physical activity levels, nutrition and stress balancing, respectively. The mean scores for the three categories were only calculated if all items were complete, no missing values were tolerated. This newly developed score for individual resources has not been tested for validity or reliability.

### Occupational health promotion programs

In the PROCARE study, standardized ergonomics and posture training, as well as back-fitness exercises were offered in nursing homes [62]. The programs were designed based on the first three steps of the BASE framework to tailor the program as much as possible [13] and were based on workplace observations and need assessments for each facility. Both interventions were conducted in a group setting (minimum of 5 persons), supervised and guided by a certified exercise scientist or physiotherapist (one instructor per training), using a standardized manual. The tasks were performed individually or in pairs, depending on the content of the training/exercise. After ten weeks of standardized ergonomics and posture training and 12 weeks of back-fitness exercises program, further demand-oriented programs were implemented according to the nurses’ preferences. Participation was assessed in the follow-up survey for the ergonomics and posture training program and the back-fitness exercises program, as both programs were offered at all study centers conducted by research staff using a standardised manual for the procedure and evaluation.

#### Ergonomics and posture training

The 10-week ergonomics and posture training program for nurses was conducted once a week for 20–30 minutes. Here, the participants learned new and beneficial techniques for their professional practice as nurses. In each unit, different work-related tasks were discussed and practiced based on workplace observations. Depending on the situation, the participants worked alone or in pairs to learn the new technique. Compensatory elements against existing occupational physical stressors were also included for each unit [76].

#### Back-fitness exercises

The 12-week back-fitness exercises program was conducted once a week for 45–60 min and was in line with the frequency, intensity, time, and type of exercise (FITT) principles [77]. It consisted of the following components: mobility, coordination training, strength training, and relaxation. The training included progressions in four-week intervals to adapt the exercise load to the increasing physical performance of the participants. The sessions were designed in a group setting. Depending on the task, the participant performed the exercise alone or in pairs.

### Procedure regarding the occupational health promotion programs

The procedure of the RCT study is reported by Otto et al. 2020 [62]. After recruitment, all participants completed the baseline questionnaires prior to joining the 10-week ergonomics and posture training and/or the 12-week back-fitness exercises. To recruit as many participants as possible, multiple dates were offered for completing the follow-up survey. In this follow-up survey, the participants had an additional question about their participation in the programs. This post measurement was done in the same way for all locations (cities) and after each occupational health program.

### Data management and analysis

The data collection and curation followed a standardized manual that covered all aspects of data management for each part of the questionnaire. For the integrated standardized tools (e.g., AVEM, SF-12) this procedure followed the original manuals. For this analysis, the dataset was first checked for missing values. Missing data were handled according to established guidelines (e.g., as outlined in the SF-12 manual), and values were imputed where appropriate.

To minimize information bias, the analysis was conducted using the “available cases” approach, based on the primary outcome participation. Furthermore, to assess the accuracy of the data, a sensitivity analysis was performed on the variable with the most missing values, which involved conducting a regression analysis without this variable. In this data set, the AVEM patterns had the most missing values. Moreover, we assessed potential selection bias by transferring the data into a “missing” and “non-missing” group. The main outcome variables were used to categorise the groups as having non-missing values (value = 0) or missing values (value = 1). We then compared the values for the main variables in these two groups with respect to age, gender, sociodemographic status and location (cities). Therefore, we conducted an independent-sample t-test.

### Statistical analysis

The overall characteristics of the study population can be found in Table 1 (*Sample description of participants with reliable data on participation in the program (n = 310)*). First, we compared the group of participants (*yes*) and non-participants (*no*) by utilizing independent t-tests controlled for multiple testing (using Bonferroni-Holm) to demonstrate the comparability of the groups with respect to occupational, demographic and individual factors. For the t-test Cohen’s d will be reported as effect size.

**Table 1:** Sample description of participants with reliable data on participation in the program (*n* = 310)

Variable	Total sample	Mean	Standard Deviation
Female	239 (77.1%)	-	-
Male	45 (14.5%)	-	-
missing gender	26 (8.4%)	-	-
Age [years]	264	42.3	11.7
FEBA - Score of physical demands	286	3.811	2.013
FEBA - Score of psychological demands	271	3.657	2.171
SSCS - Score	276	21.76	10.398
AVEM - Pattern G (%)	176	24.419	32.006
AVEM - Pattern S (%)	176	27.786	34.061
AVEM - Pattern A (%)	176	23.382	31.641
AVEM - Pattern B (%)	176	24.012	32.460
SF-12 - Score of subjective physical health components	275	45.236	8.592
SF-12 - Score of subjective psychological health components	275	45.609	10.593
Work Environment - Colleagues	274	4.263	1.120
Work Environment - Superiors	272	4.237	1.064
Work Environment - Information structure and co-determination	273	4.326	1.100
Individual Health Behavior - Physical activity	262	2.968	1.058
Individual Health Behavior - Nutrition	259	3.391	0.948
Individual Health Behavior - Stress balancing	260	2.9506	0.872

Second, we performed point serial correlations using Bonferroni-Holm adjusted significance values as a robustness check and conducted a collinearity diagnosis among all variables included in the logistic regression model. The results can be found in the supplementary material (Table S1–S4).

Third, occupational and individual factors influencing participation in occupational health promotion programs (binary dependent variable: (non-participation = 1; participation = 2) were identified using a logistic regression (odds ratio and 95% confidence interval). The significance threshold was set as *p* < 0.05. The occupational factors related to the work environment were supervisors, colleagues, information structure and co-determination [47, 52], as well as physical demands and physical load [78]. Individual factors specified were psychological demands, perceived stress, coping strategies [7, 44, 46], health status [44], and individual health behaviors related to nutrition, physical activity, and stress balancing [54]. The occupational and individual factors listed were included in the regression model using the enter method (forced entry), which jointly uses the predictors’ theoretical relevance. This simultaneous analysis of the predictors was chosen to analyses the combination of occupational and individual factors within a single model framework. Further, this approach considers the potential for synergistic effects due to interactions in the setting. This procedure is consistent with the exploratory aim in this study and the BASE framework. The model’s fit was assessed using the Hosmer-Lemeshow-test [79]. A sensitivity analysis and selectivity analysis were conducted to verify the robustness of these findings and missingness (see Table S6 and S7).

All analyses were conducted using IBM SPSS Statistics version 27 (IBM, SPSS Inc., Chicago, IL, USA).

## Results

### Participants

The characteristics captured for the final study sample are listed in Table 1.

An initial comparison between both groups, participants and non-participants, in occupational health promotion programs showed no significant group differences after controlling for multiple comparison (threshold *p* = 0.0041; see Table 2 for descriptive data). The additional robustness check (point-biserial correlation between participation and potential predictors (Table S1)) did not show any significance for participation (*p* = 0.0041).

**Table 2:** Group comparison participation and non-participation (*n* = 310)

Variable	Participation			Non-participation			Mean differences	Cohen’s d	Significance
	*n*	Mean (*Standard Deviation*)	Standard Error	*n*	Mean (*Standard Deviation*)	Standard Error			
FEBA - Score of physical demands	98	3.83 (2.05)	0.21	173	3.56 (2.24)	0.17	0.27	0.124	t = 0.992 *p* = 0.322
FEBA - Score of psychological demands	86	2.16 (1.04)	0.11	142	1.93 (1.20)	0.10	0.23	0.201	t = 1.548 *p* = 0.123
SSCS - Score	98	19.45 (8.03)	0.84	178	23.03 (11.21)	0.84	−3.58	−0.351	t = −2.769 *p* = 0.006
AVEM - Pattern G (%)	75	31.71 (33.64)	3.88	101	19.00 (29.76)	2.96	12.71	0.418	t = 2.602 *p* = 0.010
AVEM - Pattern S (%)	75	26.54 (30.25)	3.49	101	28.71 (36.76)	3.66	−2.18	−0.063	t = −0.430 *p* = 0.668
AVEM - Pattern A (%)	75	21.40 (28.94)	3.34	101	24.86 (33.57)	3.34	−3.45	−0.109	t = −0.731 *p* = 0.466
AVEM - Pattern B (%)	75	19.29 (26.97)	3.11	101	27.52 (35.73)	3.55	−8.23	−0.255	t = −1.742 *p* = 0.083
SF-12 - Score of subjective physical health components	91	46.25 (8.24)	0.86	151	44.89 (8.78)	0.71	1.36	0.158	t = 1.194 *p* = 0.234
SF-12 - Score of subjective psychological health components	91	47.73 (10.41)	1.09	151	44.14 (10.49)	0.85	3.59	0.343	t = 2.582 *p* = 0.010
Work Environment - Colleagues	99	4.35 (1.06)	0.11	175	4.21 (1.15)	0.09	0.14	0.125	t = 0.997 *p* = 0.319
Work Environment - Superiors	98	4.17 (1.00)	0.10	175	4.27 (1.10)	0.08	−0.09	−0.097	t = −0.683 *p* = 0.495
Work Environment - Information structure and co-determination	98	4.26 (0.96)	0.09	175	4.36 (1.17)	0.08	−0.10	−0.100	t = −0.737 *p* = 0.462
Individual Health Behavior - Physical activity	95	3.11 (0.98)	0.10	167	2.88 (1.09)	0.08	0.22	0.219	t = 1.594 *p* = 0.112
Individual Health Behavior - Nutrition	92	3.51 (0.92)	0.10	167	3.32 (0.95)	0.07	0.19	0.202	t = 1.534 *p* = 0.126
Individual Health Behavior - Stress balancing	94	3.03 (0.81)	0.08	166	2.90 (0.90)	0.07	0.13	0.150	t = 1.132 *p* = 0.259

*Note.* *p < 0.0042

### Influence of occupational and individual factors on participation

**Fig. 2:**
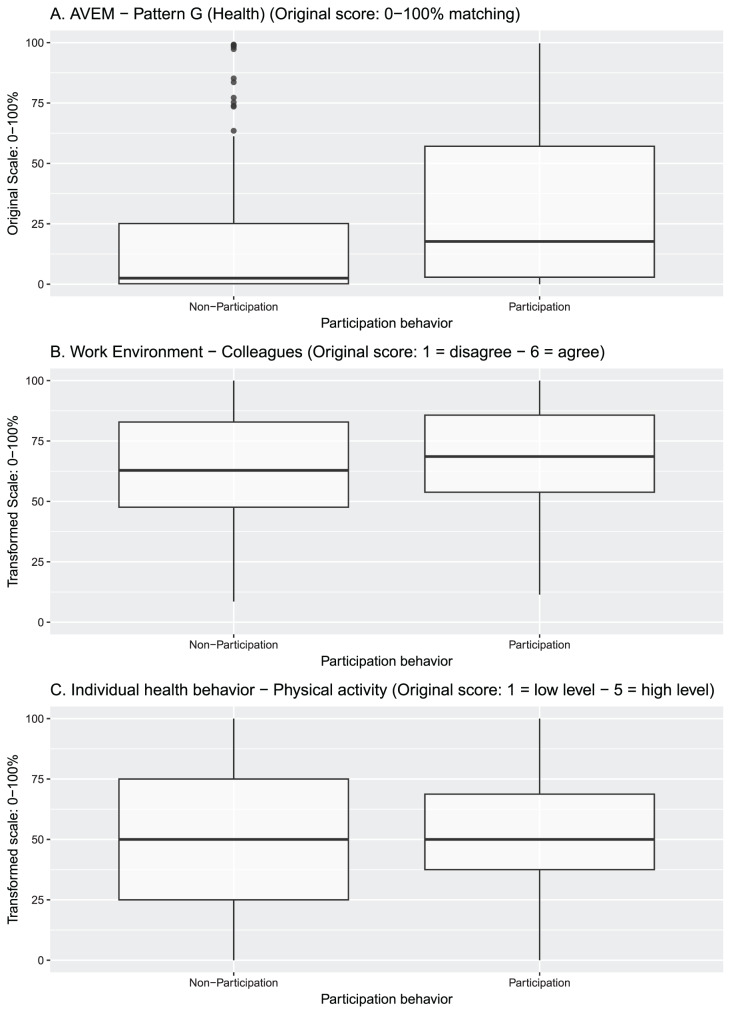
Significant predictors of participation as result of the logistic regression

The results of the logistic regression are presented in Table 3 (*Logistic Regression Model (n = 310)*). Figure 2 (*Significant predictors of participation as result of the logistic regression*) reveals a box plot of the three predictors (A-C) that are significant in the regression. Our regression model was statistically significant (*x*^*2*^ (12) = 27.547, *p* = 0.006) and correctly classified 66.7% of the cases (*Nagelkerke R*^*2*^* = 0.262*). The Hosmer-Lemeshow test exhibited a good fit for our model (*x*^*2*^ (8) = 10.648, *p* = 0.222). Collinearity check was negative for the used components in the model and is provided in the supplementary material (Table S2-4).

**Table 3:** Logistic regression model (*n* = 310)

Variable	**Scaling of the variable**^**+**^	B	*p*	Odds Ratio	95% Confidence interval	
FEBA - Score of physical demands	0–3	−0.100	0.415	0.905	[0.712	1.150]
FEBA - Score of psychological demands	0–6	−0.231	0.348	0.793	[0.489	1.287]
SSCS - Score	0–48	0.017	0.638	1.017	[0.949	1.090]
AVEM - Pattern G (%)	0–100	−0.015	0.035*	0.985	[0.971	0.999]
SF-12 - Score of subjective physical health components	0–100	−0.024	0.374	0.977	[0.927	1.029]
SF-12 - Score of subjective psychological health components	0–100	−0.051	0.087	0.951	[0.897	1.007]
Work Environment - Colleagues	1–6	−0.564	0.016*	0.569	[0.360	0.899]
Work Environment - Superiors	1–6	0.460	0.103	1.584	[0.912	2.752]
Work Environment - Information structure and co-determination	1–6	0.228	0.364	1.256	[0.768	2.055]
Individual Health Behavior - Physical activity	1–5	−0.644	0.007*	0.525	[0.328	0.842]
Individual Health Behavior - Nutrition	1–5	0.158	0.520	1.171	[0.724	1.894]
Individual Health Behavior - Stress balancing	1–5	−0.007	0.984	0.993	[0.521	1.895]

Note. *p < 0.05, Participation coding: yes = 1, no = 2, ^+^Higher scores indicate higher levels of the respective construct

We determined better coping strategies (coping pattern G (“*healthy*”), AVEM), higher rating ratings of the work environment factor colleagues (OR = 0.569; CI [0.360, 0.899]), and higher levels of physical activity (individual health behavior) (OR = 0.525; 95% CI [0.328, 0.842]) for nurses that participated in the tailored occupational health promotion programs. A statistically significant association was found for coping pattern G (“*healthy*”) showed (OR = 0.985; 95% CI [0.971, 0.999]), however, the odds ratio and the confidence interval were close to 1. The exclusion of the AVEM pattern G (sensitivity analysis), however, showed similar trends in the odds ratio values (see Table S6). The factor individual health behavior physical activity was not significant in this sensitivity analysis, but the confidence interval remains similar to that of the main analysis. Conversely, excluding AVEM resulted in a significant value for psychological health (SF-12) with a small change in confidence interval. The values for psychological demands (FEBA) showed after the exclusion a trend towards significance with also a small change in confidence interval. The workplace environment factor regarding colleagues remained significant, and the confidence intervals remained stable.

Amongst the other factors, perceived stress (SSCS), psychological and physical demands, health status, and individual health behaviors in the areas of nutrition and stress balancing revealed no consistent or statistically significant association with participation.

(see Supplement Table S7). The results of the selection analysis comparing variables having missing vs. non-missing data (see Supplement Table S7) showed that the university location factor differs significantly between these groups for all variables except work environment. Additionally, age differed significantly between the missing and non-missing groups for the individual health behavior physical activity.

## Discussion

The study aimed to analyse participation in tailored occupational health promotion programs based on occupational and individual factors of nursing staff in nursing home facilities. The results of the logistic regression showed that a higher rating of work environment with respect to colleagues and a higher level in the individual health behaviour of physical activity were associated with a higher likelihood of participating in tailored occupational health promotion programs. Interestingly, other factors such as physical load during work, perceived psychological stress/demands did not show a significant association with participation. Nevertheless, occupational and individual factors could be relevant for participation in nursing staff.

### Physical load

Contrary to previous findings [80], physical load at work was not associated with participation. This result remained stable in the sensitivity analysis when excluding the AVEM pattern G, suggesting that the findings for physical load are relatively robust. These results contrast with previous findings by Jørgensen and colleagues [79], who reported a significant association between physical load and participation in workplace health programs across a wide range of occupational settings. This discrepancy may be due to differences in work conditions and levels of physical load across industries. In contrast to Jørgensen and colleagues, our analysis was based on a relatively homogeneous sample with comparable working conditions in nursing homes, in which no association between participation and physical load was observed. Nevertheless, considering that occupational health promotion programs are supposed to serve as treatment and prevention to mitigate the identified physical demands and strains, a higher participation among those experiencing greater physical loads is essential. Future studies could further investigate under which conditions physical load may change participation.

### Psychological demands, stress, and coping strategies

Unexpectedly, our analysis revealed no association between participation and examined psychological demands (pressure to perform, deadline pressure, and time pressure) or perceived stress. A statistically significant association with participation was found for a favourable coping strategy, although the practical relevance appeared limited considering the OR close to 1, indicating limited practical relevance. The sensitivity analysis excluding the AVEM pattern G showed that the association with participation changed for psychological health and demands. The SF-12 for psychological health parameter became significant. The FEBA score for psychological demands is showing a trend toward significance. For both parameters the confidence interval showed a difference compared to the main analysis.

Psychological stress is described as an interplay between various stressors and a person’s resilience [81, 82]. Although no statistical evidence of multicollinearity was observed, conceptual overlap between some predictors (e.g. psychological demands and coping strategies, both reflecting aspects of work-related stress) may have contributed to an attenuation of the individual effects in the regression model.

In addition to protective health resources, the individual perception of stress is decisive, and the different coping types seem to vary in their perceptions of stress-related burdens [43]. A high variability in stress perception of the participants may have contributed to non-significant findings for psychological components for participation. The heterogeneity in the results could be caused by differences in stress quantities (duration, intensity of stressors), as well as by working conditions such as shift work. A detailed analysis of health-affecting subcomponents of psychological stressors, like personal traits or single personal or occupational stressors, may help to better understand participation in occupational health promotion programs in future studies. Additionally, other clinical settings and programs using participation as a parameter could provide insight into the relationship with stress.

### Work environment

As expected, and in line with previous research, our model showed that a better work environment with respect to colleagues was associated with a higher likelihood of participating in tailored occupational health promotion programs [83]. The robustness of this association was supported by a similar trend in the sensitivity analysis.

Similarly, Hung and colleagues have shown that nurses with the highest participation in occupational health promotion programs reported better teamwork than those with the lowest participation [83]. The significant association between the predictor colleagues and participation is consistent with previous findings by Burke et al. [84]. Their meta-analysis suggested that the opportunity to interact with each other may be associated with higher participation in exercise programs [84].

Other workplace factors (superiors, information structure and co-determination) were not significantly associated with participation mirroring findings from a survey of occupational health promotion and management experts [85]. Other studies in the healthcare sector have found both the significance of leadership in shaping work experiences and addressing mistreatment [86, 87] and that improving job satisfaction and sharing information may improve participation [83, 88]. Additionally, previous studies reported that an overabundance of information in the workplace represents a barrier to receiving messages regarding occupational health promotion programs [83, 88]. Further, information structure and co-determination have known effects on mental and physical health, job turnover, and quality of care, potentially impacting participation. This suggests a possible indirect association between work environment and participation that could be investigated by future research [47, 89, 90]. Moreover, these factors might be considered prior to the implementation of health promotion programs as potentially to facilitating participation. In addition, it appears to be a challenge to map the implicit structures of each organization’s work environment and corporate culture (e.g., expectations and indirect communications), as this may also be associated with the success of occupational health promotion programs [91, 92]. Whether team-building programs within a continuous participatory process could increase participation remains to be tested in subsequent programs.

### Individual health behavior

Our analysis revealed that the higher individuals’ physical activity behavior was associated with a higher likelihood of participation. This significant association disappeared in the sensitivity analysis, although the OR trend remained stable, suggesting limited robustness for physical activity. The observation of our main analysis is in line with our expectation and aligns with related studies that have previously identified an association between high leisure-time physical activity and participation in organized activities [93, 94]. Individuals with a more active lifestyle may also be more likely to participate in occupational health promotion programs. From a behavioral uptake perspective, prior engagement in occupational health programs and intrinsically motivated health behaviors are associated with a higher likelihood of participating in additional offered programs [95]. This is also consistent with evidence linking higher baseline physical activity to greater participation in tailored physical activity interventions [96]. In other occupational settings (employees at the university), Abraham and colleagues found that people who had already participated in sports in the past were more likely to register for the exercise program. In addition, time availability/cost (how long it takes to get there) and personal preferences were identified as factors for registering in an exercise program [97].

However, it is important to note that nurses’ health behaviors and resources are also related to other factors, such as lower health status and mental and physical health, which have already been shown in a previous study with the PROCARE data set [44]. These additional components may indirectly influence participation in the occupational health promotion programs examined in the current study. Prior research has shown that the perceived importance of specific health-related behaviors (e.g., fruit and vegetable consumption) can also affect participation in corresponding occupational health promotion programs (e.g., healthy eating interventions) [98, 99]. However, positive values of individual health behavior related to nutrition were not associated with participation in our study.

Similar to the nutrition-related behavior, stress balancing was not associated with participation. It has previously been shown that individuals exhibiting more favorable health behaviors in general are more likely to participate in other prevention and health-related offerings [100]. However, this general carry-over effect of health behavior was not confirmed within our study either. These findings suggest that engagement in one domain of health behavior (e.g., stress balancing) does not necessarily translate into participation in programs addressing a different domain (e.g., ergonomics training).

Despite efforts to achieve high participation, participation varied considerably in our study (participation in our analysis: 39,4%). A scoping review identified a variation in participation rates of 7–100% across different programs in the USA [101]. While our assumptions are grounded in the German healthcare system, they may therefore have some relevance for other settings. The sample consisted predominantly of women (here: pink-collar workers), precluding meaningful gender-stratified analyses. This imbalance may also have influenced participation. In contrast, studies conducted in male-dominated occupational groups (e.g., blue-collar workers) might yield different patterns and help to clarify potential gender effects.

Within the PROCARE program, participants were asked if they would like to join a tailored occupational health promotion program and what type of exercise they would like to engage in (participative approach) [62]. The initial interest was high (more than 80%) and program components with the highest ratings were back fitness and ergonomic posture training. Accordingly, the final occupational health promotion program (back-fitness exercises program, ergonomics and posture training) followed the participants’ wishes captured in the participatory approach, as specified in the BASE framework [13]. We anticipated that using this participatory approach when designing and implementing a tailored occupational health promotion program may be associated with higher participation and higher identification or satisfaction with the programs, as shown in previous studies, regardless of individual health behavior.

The BASE framework provided a conceptual basis for examining participation as an outcome [13, 14]. Additional factors and processes (unconscious habits or attitudes) that were not captured in this study may also influence participation [102–104]. Especially, factors like personality traits may affect the barriers and resources among nursing staff [7]. Using a participatory approach alone may not be sufficient to support participation as personal characteristics may also affect people’s willingness to participate [96]. Future research on participation should also consider these traits.

Moreover, participation should be further examined in different settings using occupational and individual factors such as those explored in this study. Also, self-efficacy and social support in the occupational group might influence participation. The identified association between self-efficacy and the participation in health promotion activities of nursing staff suggests that the aims and approaches may require further considerations in health promotion [7]. It could be relevant to consider self-efficacy and social support more strongly in the design of occupational health promotion programs [105, 106].

Considering the implementation process, participation could be an indicator for success rather than an individual outcome [107, 108]. For the BASE framework and its implementation into workplace health promotion the study results suggest, that the implementation process needs more effort than a participatory planning of desired interventions. An integration of implementation frameworks (e.g. European Good Practice, concepts of Mastenbroeck and colleagues), could help to gain are a more successful and economical implementation [109–111]. However, such concepts may challenge standardization within randomized controlled trials and other future studies. In summary, these different factors together with different theoretical and conceptual considerations highlight the complexity of individual, social, occupational and implementation-related factors, which could not fully be captured with this study.

## Limitations

Despite the advantage of having a representative cohort of nursing staff from all over Germany in this study, several potential limitations should be considered.

### Sample size, missing data, and selection bias

The substantial reduction from over 3,000 eligible participants to around 300 analysed cases indicates a risk of missing data and selection bias. Although selectivity analyses suggested that data were largely missing completely at random, observed differences by location (cities) and age point to some sample imbalance, which may also reflect institutional differences. In addition, language and comprehension barriers particularly in a workforce with a high proportion of nursing staff with a migrant background in some German areas may have further contributed to missing data and selective participation [112]. Similarly, it is possible that stress factors were underestimated in the regression, as it is less likely that additional data would be provided in this case. This bias should be considered when interpreting the results and suggests that participation may be skewed towards well-connected and more active nursing staff. This could be circumvented by a continuous attendance measurement within the programs and addressing explicitly participants hard to approach.

### Statistical limitations and model stability

The relatively large number of predictors (*N* = 12) used simultaneously in relation to the sample size increased the risk of overfitting and unstable estimates, as reflected in sensitivity analyses and potential overlap between predictors. In addition, certain variables, such as stress-related factors, may be underestimated due to reporting tendencies. Consequently, the findings should be interpreted as exploratory and hypothesis-generating rather than causal. Future analyses should consider a more parsimonious or hierarchical modelling approach with a reduced number of predictors based on these current findings and further occupational or individual considerations.

### Measurement and information bias

Methodological limitations should also be acknowledged. In particular, participation was assessed dichotomously, which represents an important limitation, as it does not capture fluctuations in participation or participation over time and may introduce measurement and misclassification bias. However, the sensitivity analysis, excluding the parameter with the most missing values (AVEM) suggests the robustness of the results of the available cases analysis. In addition, participants with missing follow-up responses were classified as non- participation, which may have led to misclassification by conflating non-response with non-participation. Furthermore, the work environment questionnaire, although expert-reviewed, has not undergone formal reliability testing.

Future studies should incorporate continuous measures of participation rate or respective dynamics (e.g. frequency, intensity) and consider hard-to-reach groups. Furthermore, this secondary analysis relied on the data structure of the PROCARE project. The use of standardized scales measuring the same dimensions would facilitate more uniform evaluation in future research. Nevertheless, differences between the scales did not affect our results or their interpretation. Despite the measurement and information biases mentioned, the study can provide first findings that allow for a closer examination of participation in the occupational setting.

### Program implementation and contextual variability

Contextual and implementation-related factors may also have influenced participation. Differences in program promotion across universities and facilities, as well as variation in institutional characteristics such as size and staff density, could have affected how many employees were informed about and able to participate in the programs (See Table S5). Moreover, program characteristics including session duration, timing, and compatibility with shift systems may have acted as barriers or facilitators for both the individual and organizational factors.

### Validity and generalizability

The reliance on the use of an extensive survey administered during working hours may have further impacted response quality, but at the same time allowed us to reach the target group. While the multicenter design enhances external validity within German nursing home settings, generalizability to other healthcare contexts, such as hospitals, remains limited due to the relative homogeneity of the sample. Internal validity should be interpreted with caution given the outlined methodological constraints. Finally, participation may also have been affected by the working conditions during the COVID-19 pandemic, including hygiene restrictions and increased time pressure; however, the specific impact of these factors could not be assessed in the present analysis. Nevertheless, this study used a comprehensive multicentre German wide RCT dataset that followed prevention guidelines and obtained results on participation and their potential predictors in a homogenous occupational setting.

## Conclusion

To the best of our knowledge, this study is the first to investigate determinants for participation in tailored occupational health promotion programs conducted in nursing homes among nursing staff. Individual health behavior (physical activity) and work environment (colleagues) were positively associated with participation. These factors could be considered when designing and implementing occupational health promotion programs as potential factors relevant to participation. Our findings are consistent with previous research in other occupational settings showing participation in occupational health promotion programs to be associated with multiple factors such as work environment and health behavior. The results highlight the importance of participation analysis as a key component in the evaluation of occupational health promotion programs and contribute to identifying what may be relevant for their successful implementation. Future research could further investigate these relationships using more detailed participation data and in a more diverse sample in prospective study designs. Also related components, such as motivation, personal relevance of the topic, and organizational support and context, might be depicted in greater detail in further analysis.

## Electronic supplementary material

Below is the link to the electronic supplementary material.

Supplementary Material 1

## Data Availability

Data utilized in this study will be made available by the authors based on a reasonable request. Regarding the PROCARE study, the data set was requested from the PROCARE consortium after the project ended. Data may only be provided after researchers seeking data must state their future objectives and discuss the approach with the PROCARE project leaders.

## Footnotes

1 The questionnaire is available in German on request from the authors.

2 The questionnaire is available in German on request from the authors.
