# Supplementary material for: Occupational and personal factors contributing to participation in occupational health promotion programs for employees in nursing home facilities: a secondary analysis of the PROCARE study
Source: BMC Nurs. 2026 Jul 9;25:610. doi: 10.1186/s12912-026-04973-6 (PMC13352799; doi:10.1186/s12912-026-04973-6)
Supplement: Supplementary file 1 — Supplementary Material 1 [file 12912_2026_4973_MOESM1_ESM.docx]

Supplementary Material

**Table S1 Correlation table**

| Variable | Point biserial correlation | *p* value |
| --- | --- | --- |
| SSCS-Score | .165 | .006 |
| AVEM - Pattern G (%) | -.197 | .009 |
| AVEM - Pattern S (%) | .032 | .667 |
| AVEM - Pattern A (%) | .054 | .476 |
| AVEM - Pattern B (%) | .126 | .096 |
| SF-12 - Score of subjective physical health components | -.077 | .234 |
| SF-12 - Score of subjective psychological health components | -.164 | .010 |
| FEBA - Score of physical demands | -.059 | .334 |
| FEBA - Score of psychological demands | -.099 | .137 |
| Work Environment - Colleagues | -.060 | .319 |
| Work Environment - Superiors | .042 | .495 |
| Work Environment - Information structure and co-determination | .045 | .462 |
| Individual Health Behavior - Physical activity | -.098 | .112 |
| Individual Health Behavior - Nutrition | -.095 | .126 |
| Individual Health Behavior - Stress balancing | -.070 | .259 |

*Note.* **p* < .0042

**- Table S2 Correlations between main values of the regression model (Spearman-Rho)**

|  | SSCS - Score | AVEM - Pattern G | SF-12 - Score of subjective physical health components | SF-12 - Score of subjective psychological health components | FEBA - Score of physical demands | FEBA - Score of psychological demands | Work Environment - Colleagues | Work Environment - Superiors | Work Environment - Information structure and co-determination | Individual Health behavior - Physical activity | Individual Health Behavior- Nutrition | Individual Health Behavior – Stress balancing |
| --- | --- | --- | --- | --- | --- | --- | --- | --- | --- | --- | --- | --- |
| SSCS - Score | 1.0000 |  |  |  |  |  |  |  |  |  |  |  |
| AVEM - Pattern G | -0.210** | 1.0000 |  |  |  |  |  |  |  |  |  |  |
| SF12 - Score of subjective physical health components | -0.313** | 0.029 | 1.0000 |  |  |  |  |  |  |  |  |  |
| SF12 - Score of subjective psychological health components | -0.660** | 0.375** | 0.134* | 1.0000 |  |  |  |  |  |  |  |  |
| FEBA - Score of physical demands | 0.350** | -0.166* | -0.277** | -0.223* | 1.0000 |  |  |  |  |  |  |  |
| FEBA - Score of psychological demands | 0.421** | -0.168* | -0.219** | -0.300** | -0.450** | 1.0000 |  |  |  |  |  |  |
| Work Environment - Colleagues | -0.289** | 0.123 | 0.161* | 0.131* | -0.188** | -0.115 | 1.0000 |  |  |  |  |  |
| Work Environment - Superiors | -0.431** | 0.340** | 0.233** | 0.341** | -0.237** | -0.207** | 0.547** | 1.0000 |  |  |  |  |
| Work Environment - Information structure and co-determination | -0.286** | 0.282** | 0.234** | 0.290** | -0.285** | -0.228** | 0.348** | 0.619** | 1.0000 |  |  |  |
| Individual Health Behavior - Physical Activity | -0.159* | -0.112 | -0.122 | 0.177** | -0.062 | -0.040 | -0.018 | 0.026 | 0.093 | 1.0000 |  |  |
| Individual Health Behavior - Nutrition | -0.240** | -0.094 | 0.149* | 0.213** | -0.164** | -0.079 | 0.086 | 0.032 | 0.024 | 0.310** | 1.0000 |  |
| Individual Health Behavior - Stress balancing | -0.542** | -0.014 | 0.360** | 0.508** | -0.293** | -0.286** | 0.222** | 0.299** | 0.275** | 0.287** | 0.426** | 1.0000 |

*Note.* **p* < .05. ***p* < .01. ****p* < .001

**Table S3 Collinearity diagnosis of the values of the regression model**

| Variance Proportions | | | | | | | | | | | | | | | |
| --- | --- | --- | --- | --- | --- | --- | --- | --- | --- | --- | --- | --- | --- | --- | --- |
| Dimension | One values | Condition index | Intercept | SSCS - Score | AVEM - Pattern G | SF-12 - Score of subjective physical health components | SF-12 - Score of subjective psychological health components | FEBA - Score of physical demands | FEBA - Score of psychological demands | Work Environment - Colleagues | Work Environment - Superiors | Work Environment - Information structure and co-determination | Individual Health Behavior - Physical activity | Individual Health Behavior - Nutrition | Individual Health Behavior - Stress balancing |
| 1 | 11.406 | 1.000 | .00 | .00 | .00 | .00 | .00 | .00 | .00 | .00 | .00 | .00 | .00 | .00 | .00 |
| 2 | .687 | 4.076 | .00 | .01 | .41 | .00 | .00 | .02 | .01 | .00 | .00 | .00 | .00 | .00 | .00 |
| 3 | .362 | 5.613 | .00 | .02 | .31 | .00 | .00 | .08 | .04 | .00 | .00 | .00 | .01 | .00 | .01 |
| 4 | .123 | 9.648 | .00 | .07 | .01 | .01 | .00 | .27 | .04 | .03 | .01 | .01 | .23 | .01 | .01 |
| 5 | .111 | 10.141 | .00 | .09 | .07 | .00 | .00 | .33 | .01 | .02 | .03 | .01 | .22 | .04 | .00 |
| 6 | .089 | 11.296 | .00 | .15 | .00 | .00 | .01 | .06 | .71 | .01 | .00 | .01 | .03 | .02 | .01 |
| 7 | .068 | 12.936 | .00 | .03 | .01 | .02 | .01 | .01 | .12 | .04 | .02 | .02 | .47 | .17 | .08 |
| 8 | .042 | 16.471 | .00 | .00 | .00 | .10 | .00 | .02 | .02 | .15 | .01 | .02 | .00 | .55 | .17 |
| 9 | .036 | 17.861 | .00 | .00 | .10 | .02 | .06 | .01 | .00 | .25 | .00 | .44 | .00 | .04 | .15 |
| 10 | .031 | 19.233 | .00 | .18 | .01 | .48 | .08 | .03 | .01 | .02 | .00 | .00 | .01 | .06 | .25 |
| 11 | .023 | 22.457 | .01 | .00 | .05 | .03 | .55 | .01 | .01 | .09 | .15 | .11 | .00 | .06 | .26 |
| 12 | .019 | 24.354 | .00 | .06 | .03 | .00 | .05 | .06 | .01 | .32 | .76 | .35 | .01 | .01 | .05 |
| 13 | .004 | 52.551 | .99 | .39 | .00 | .35 | .23 | .10 | .02 | .07 | .01 | .03 | .01 | .03 | .00 |

**Table S4 Variance inflation factors of the values of the regression model**

| Value | Tolerance | VIF |
| --- | --- | --- |
| SSCS - Score | .380 | 2.631 |
| AVEM - Pattern G | .709 | 1.418 |
| SF-12 - Score of subjective physical health components | .738 | 1.356 |
| SF-12 - Score of subjective psychological health components | .494 | 2.420 |
| FEBA - Score of physical demands | .368 | 2.714 |
| FEBA - Score of psychological demands | .512 | 1.952 |
| Work Environment - Colleagues | .597 | 1.676 |
| Work Environment - Superiors | .420 | 2.382 |
| Work Environment - Information structure and co-determination | .872 | 1.749 |
| Individual Health Behavior - Physical activity | .864 | 1.158 |
| Individual Health Behavior - Nutrition | .771 | 1.297 |
| Individual Health Behavior - Stress balancing | .557 | 1.795 |

**Table S5 Overview of nursing staff and subjects were part of the PROCARE interventions**

| Locations | Nurses in nursing home facilities (in total) | Participants in the intervention (percentage) |
| --- | --- | --- |
| Location 1 | 631 | 340 (53,9 %) |
| Location 2 | 381 | 87 (22,9 %) |
| Location 3 | 553 | 153 (27,7 %) |
| Location 4 | n/a | 270 (n/a) |
| Location 5 | 453 | 118 (26 %) |
| Location 6 | 646 | 189 (29,3 %) |
| Location 7 | 530 | n/a |
| Location 8 | 429 | 105 (24,5 %) |
| **In total** | **3623** | **1265 (34,9 %)** |

**Table S6 Sensitivity analysis: Logistic regression without the AVEM (most missing values)**

| **Variable** | **B** | **p** | **Odds Ratio** | **95% Confidence interval** | |
| --- | --- | --- | --- | --- | --- |
| SSCS - Score | .029 | .294 | 1.029 | [.975 | 1.086] |
| SF-12 - Score of subjective physical health components | -.039 | .088 | .962 | [.920 | 1.006] |
| SF-12 - Score of subjective psychological health components | -.060 | .012* | .942 | [.899 | 0.987] |
| FEBA - Score of physical demands | -.118 | .226 | .889 | [.735 | 1.076] |
| FEBA - Score of psychological demands | -.358 | .056 | .699 | [.485 | 1.009] |
| Work Environment - Colleagues | -.426 | .023* | .653 | [.453 | .942] |
| Work Environment - Superiors | .318 | .176 | 1.374 | [.867 | 2.177] |
| Work Environment - Information structure and co-determination | .227 | .272 | 1.255 | [.837 | 1.884] |
| Individual Health Behavior - Physical activity | -.294 | .106 | .745 | [.522 | 1.064] |
| Individual Health Behavior - Nutrition | .105 | .605 | 1.111 | [.746 | 1.654] |
| Individual Health Behavior - Stress balancing | .047 | .863 | 1.048 | [.618 | 1.777] |

*Note. *p<.05, Participation coding: yes=1, no=2*

**Table S7 Selectivityanalysis of the main predictors of the model**

| **Group variable** | **Testvariables** | **T** | **p value (unequal)** | **Cohen’s d** |
| --- | --- | --- | --- | --- |
| SSCS – Score – missing | Age | .349 | .735 | .125 |
|  | Gender | -.726 | .380 | -.214 |
|  | Socioecological status | -1.749 | .075 | -.325 |
|  | University standpoint | 5.559 | .001* | .606 |
| AVEM - Pattern G (%) – missing | Age | -1.419 | .153 | -.181 |
|  | Gender | -1.125 | .262 | -.133 |
|  | Socioecological status | -.849 | .405 | -.096 |
|  | University standpoint | 7.843 | .001* | .860 |
| SF-12 - Score of subjective physical health components – missing | Age | -1.138 | .260 | -.202 |
|  | Gender | -1.008 | .261 | -.162 |
|  | Socioecological status | -.174 | .862 | -.026 |
|  | University standpoint | 3.444 | .001* | .413 |
| SF-12 - Score of subjective psychological health components – missing | Age | -1.138 | .260 | -.202 |
|  | Gender | -1.134 | .261 | -.162 |
|  | Socioecological status | -.174 | .862 | -.026 |
|  | University standpoint | 3.444 | .001* | .413 |
| FEBA - Score of physical demands – missing | Age | .625 | .542 | .221 |
|  | Gender | .851 | .407 | .265 |
|  | Socioecological status | -.673 | .504 | -.131 |
|  | University standpoint | 4.390 | .001* | .532 |
| FEBA - Score of psychological demands – missing | Age | -.632 | .530 | -.101 |
|  | Gender | -.417 | .667 | -.062 |
|  | Socioecological status | -1.595 | .113 | -.195 |
|  | University standpoint | 8.703 | .001* | .839 |
| Work Environment - Colleagues – missing | Age | -.523 | .623 | -.168 |
|  | Gender | -.165 | .871 | -.045 |
|  | Socioecological status | -.461 | .647 | -.086 |
|  | University standpoint | 1.671 | .101 | .234 |
| Work Environment - Superiors – missing | Age | -.331 | .746 | -.100 |
|  | Gender | -.401 | .693 | -.096 |
|  | Socioecological status | -.790 | .434 | -.143 |
|  | University standpoint | 1.603 | .114 | .216 |
| Work Environment - Information structure and co-determination – missing | Age | -.525 | .610 | -.168 |
|  | Gender | -1.377 | .186 | -.265 |
|  | Socioecological status | -.870 | .389 | -.155 |
|  | University standpoint | 1.140 | .259 | .169 |
| Individual Health Behavior - Physical activity – missing | Age | -2.930 | .007* | -.654 |
|  | Gender | .913 | .369 | .218 |
|  | Socioecological status | -1.172 | .246 | -.207 |
|  | University standpoint | 3.860 | .001* | .477 |
| Individual Health Behavior – Nutrition – missing | Age | -2.445 | .021* | -.547 |
|  | Gender | .676 | .504 | .147 |
|  | Socioecological status | -1.405 | .165 | -.239 |
|  | University standpoint | 3.106 | .003* | .401 |
| Individual Health Behavior - Stress balancing – missing | Age | -1.688 | .103 | -.404 |
|  | Gender | .754 | .456 | .169 |
|  | Socioecological status | -1.092 | .279 | -.187 |
|  | University standpoint | 3.188 | .002* | .410 |

*Note. *p<.05*
